# Supplementary material for: Women’s lived experiences of preterm birth and neonatal care for premature infants at a tertiary hospital in Ghana: A qualitative study
Source: PLOS Glob Public Health. 2022 Dec 1;2(12):e0001303. doi: 10.1371/journal.pgph.0001303 (PMC10022110; doi:10.1371/journal.pgph.0001303)
Supplement: S1 File — (DOCX) [file pgph.0001303.s001.docx]

**S1 File: Interview guide for mothers who experienced preterm birth**

1. Can you tell me something about your pregnancy from the time you got pregnant to the time you came to Korle-Bu?
2. Have you had any problems/complications during this pregnancy?
3. Did you have any issue that bothered you, like financial problems, marital problems, job issues or living conditions? Probe further.
4. In your opinion, do you think the time you delivered was normal or you think your delivery has occurred too early. Can you explain this point?
5. How long did you have to wait before you were attended to?
6. What complications of preterm birth do you know of?
7. Can you mention you some of the causes of preterm birth?
8. How did your admission affect you and your family?
9. How would describe the interaction you had with the doctors/nurses?
10. What is the most difficult aspect of the care you received here?
11. Did you experience any disrespectful care, eg verbal abuse like shouting at you, physical abuse?
12. Do you think there were adequate number of doctors and nurses to attend to you and all the other patients at the ward?
13. Did you received the care you expected from the doctors and nurses?
14. What challenges did you experience when you were on admission?
15. In future would you prefer to be cared for here or elsewhere? Why?
16. Do you have any recommendations (to the doctors, nurses, administrators) to improve the quality of care of women who present with similar conditions?
17. What aspects of treatment as a patient do you find important or helpful?
18. Do you think something could have been done to prevent the preterm birth you have experienced?
19. When do you feel you have received good care?
20. When do you feel you have not received good care?
