# Supplementary material for: Women’s lived experiences of preterm birth and neonatal care for premature infants at a tertiary hospital in Ghana: A qualitative study
Source: PLOS Glob Public Health. 2022 Dec 1;2(12):e0001303. doi: 10.1371/journal.pgph.0001303 (PMC10022110; doi:10.1371/journal.pgph.0001303)
Supplement: Attachment — Submitted filename: Response to reviewer comments_7July2022.docx [file pgph.0001303.s002.docx]

Dear Editor,

Kindly find below point-by-point response to the reviewers’ comments on our our manuscript titled **“Women’s lived experiences of preterm birth and neonatal care for premature infants at a tertiary hospital in Ghana: A qualitative study”***.*

We would like to thank the peer reviewers and the editors for their comments, which have been very helpful in revising the manuscript. We have made the necessary revisions to our manuscript in line with recommendations suggested by the Reviewers/Editors.

We hope that our responses are satisfactory for acceptance.

We look forward to hearing from you.

Sincerely,

Dr Kwame Adu-Bonsaffoh

Corresponding author

Journal Requirements:

1. In the online submission form, you indicated that "The transcripts for this qualitative study will be made available upon reasonable request from the corresponding author". All PLOS journals now require all data underlying the findings described in their manuscript to be freely available to other researchers, either 1. In a public repository, 2. Within the manuscript itself, or 3. Uploaded as supplementary information.

This policy applies to all data except where public deposition would breach compliance with the protocol approved by your research ethics board. If your data cannot be made publicly available for ethical or legal reasons (e.g., public availability would compromise patient privacy), please explain your reasons by return email and your exemption request will be escalated to the editor for approval. Your exemption request will be handled independently and will not hold up the peer review process, but will need to be resolved should your manuscript be accepted for publication. One of the Editorial team will then be in touch if there are any issues.

**Response: We thank you for this editorial policy on data availability. This a qualitative study and all the participants’ responses are included in the manuscript**

2. We have noticed that you have uploaded Supporting Information files, but you have not included a list of legends. Please add a full list of legends for your Supporting Information files after the references list.

**Response: We have provided list of legends for the uploaded supporting files**

Additional Editor Comments (if provided):

Thank you for submitting your manuscript. The work shows promise, but significant revisions are needed to make the manuscript ready for publication. Particular attention should be paid to the care context in Ghana and in developing a theoretical framework to position the results.

**Response: We thank you for this important comment. We have revised the Introduction and Methods to clearly indicate the Ghanaian context and improve the rationale of the study.**

Reviewers' comments:

**5. Review Comments to the Author**

Please use the space provided to explain your answers to the questions above. You may also include additional comments for the author, including concerns about dual publication, research ethics, or publication ethics. (Please upload your review as an attachment if it exceeds 20,000 characters)

Reviewer #1: The manuscript is generally well written and touches on the subject of prematurity which is an old problem with renewed concern following the increasing use of assisted reproductive technology in developing countries. However, these minor changes need to be effected.

1. Title: There should be a inserted before the tertiary.

**Response: We thank the reviewer pointing out this omission. We have revised the title to as recommended**

2. Abstract: well written

**Response: We thank the reviewer for the commendation. No response is required**

3. Introduction: well written

**Response: We thank the reviewer for the commendation. No response is required**

4. Methods: Doing focus group discussions for the mothers to further explore their experiences in a group setting would have further enhanced the quality of your work.

**Response: We thank the reviewer for this positive comment. We agree that inclusion of Focus Group Discussion would have provided more varied experiences from the mothers and improved the results. We have included the non-inclusion of FGD as a limitation of the study**

As I understand, maternal and new born health are covered under the Ghana NHIS. How come mothers are still complaining of financial burden?

**Response: We appreciate this important comment from reviewer. In Ghana, the NHIS does not cover the full cost of maternal health and mothers complain frequently about this issue. We have indicated this in the manuscript**

Key informant interviews of healthcare personnel and key policy makers could have helped unravel some of these challenges. If it is possible to be done, the authors should consider doing so, otherwise this should be captured also as a limitation in the study.

**Response: We thank the reviewer for this important comment. Unfortunately, Key informant interviews of healthcare personnel and key policy makers are not included in this study. We have captured this as a limitation of the study**

5. The result is well written.

**Response: We thank the reviewer for the commendation. No response is required**

cord and skin cleansing with chlorhexidine

6. The authors must get more literature to explain why cost should be a great burden in a country where maternal and neonatal health is reported to be fully covered on the insurance. Is this observation reported in other countries? Instead of recommending for a full coverage, the authors should rather call for a probe as to why the mismatch between policy and practice and use other best practices elsewhere to suggest a workable solution.

**Response: We thank the reviewer for this important comment on the coverage issues of NHIS in the country. We have provided more literature and clarification concerning the NHIS issues in our country and related to other countries in SSA.**

7. General comments: Kindly read through the work thoroughly and correct all typos, spacing and complete your sentences. For eg line 90 is not completed.

**Response: We have revised the manuscript and corrected all the typos and spacing issues as recommended by the reviewer.**

8. No commendation for the NICU staff. Are they part of the Obgyn dept?

**Response: We thank the reviewer for enquiring about the NICU staff. Although the NICU at the study site is located in the Maternity Block, it is technically not controlled by the OBGYN Department but by the Department of Child Health. However, the two department work together on issues related to Neonatal Health**

Reviewer #2: This research reports the hospital experience of mothers with preterm birth in Ghana. Using qualitative methodology, four themes were identified: knowledge of the risk of preterm birth and its complications, experience of newborn admission to the neonatal intensive care unit, financial burden to families of preterm birth, and recommendations to improve the quality of care.

1. The manuscripts reports the universal experiences of families after preterm birth and NICU admission, regardless of location of birth: fear regarding uncertainty of outcome, maternal emotional distress at separation from the newborn, and financial burden, regardless of health insurance status. There have been systematic reviews of both quantitative and qualitative data on parents experiences of NICU admission. The unique aspect of the paper is how these universal themes are manifest in Ghana.

**Response: We thank the reviewer for this commendation. No response is required**

General comments:

2. Additional context is needed about the health system in Ghana to appreciate the study results. Readers may not understand that families play a critical role in treatment plans for both hospitalized mothers and newborns by purchasing prescribed drugs separately and delivering them to the bedside (at great financial burden for low income families), and physically obtaining and delivering laboratory results to doctors ( increasing both psychological and physical stress on postpartum women).

**Response: We have provided additional context about the clinical care for patients in the health system in Ghana as recommended. We have related the role of family members in the care for patients on hospital admission**

3. Additional information is also needed about the average income of families in US dollars (since the authors use this conversion metric) so that reader from HICs can understand the financial burden of preterm birth in this LMIC. Issues of insurance of coverage and healthcare cost for ill and preterm/LBW newborns in Ghana have previously been published (Enweronu-Laryea CC, Andoh HD, Frimpong-Barfi A, et al. Parental costs for in-patient neonatal services for perinatal asphyxia and low birth weight in Ghana. PLoS ONE 2018; 555 13: 1–14).
**Response: We Thank the reviewer for this comment on issues of insurance coverage and healthcare cost. We have already cited the paper by Enweronu-Laryea et al in the discussion. We have added more from the same paper based on your recommendation. We have also included average income of households as recommended by the reviewer**

4. Throughout the manuscript understanding of the maternal experience of care and recommendations for improvement is sometimes stated as “improving clinical care for preterm infants” (line 398). This may be a matter of translation but needs to be corrected; the manuscript needs editing for meaning in English.

**Response: We thank the reviewer for pointing this grammatical error. We have made the required correction in the sentence**

Methods:

5. Participants are labelled as a convenience sample, but the description sounds as if purposive sampling was used (lines 125-126:”…. the aim of achieving varied obstetric history, age and socio-economic characteristics.”). This should be clarified with a description of how screening for representative characteristics was achieved. If a broadly representative sample is achieved should be reported in the results.

**Response: We thank the reviewer for this important comment. We agree with comment on purposive sampling. We have revised the Methods to reflect this correction**

6. The interview asked a leading question concerning finances (Did you have any issue that bothered you, like financial problems, marital problems, job issues or living conditions?) which may have influenced the results, but I think there were other open ended questions that gave participants an opportunity to expand on the issue of financial burden.

**Response: We thank the reviewer for this relevant comment. We agree that the use of the closed ended question (as stated above) may have influenced the results. However, the question were probed further which elicited deeper responses concerning the challenges they faced.**

7. Content analysis was appropriate.

**Response: We thank the reviewer for this positive comment. No response is required**

Results:

8. Results are straightforward and illustrate some of the unique stressors families face after preterm birth. The finding that women are not allowed to leave the hospital until the bill is paid even though medically cleared has not been previously reported that I have seen. This was certainly my experience with pediatric patients in Kenya, too.

**Response: We thank the reviewer for the commendation. No response is required**

Discussion:

9. The Discussion is the strongest part of the paper but would benefit from more context about the health system in Ghana presented earlier in the paper. While I find these data fascinating, I am not sure that they are unique enough to warrant publication.

**Response: We thank the reviewer for the comment on improving the discussion section of the paper. We have revised the Discussion section by including the Ghanaian context as recommended**
